# Activated brown adipose tissue and its relationship to adiposity and metabolic markers: an exploratory study

**DOI:** 10.1080/21623945.2020.1724740

**Published:** 2020-02-11

**Authors:** Malini Soundarrajan, Jie Deng, Mary Kwasny, Nicholas C. Rubert, Paige C. Nelson, Dalya A. El-Seoud, Lewis Landsberg, Lisa M. Neff

**Affiliations:** a Division of Endocrinology, Metabolism and Molecular Medicine, Northwestern University, Feinberg School of Medicine, Chicago, IL, USA; b Department of Diagnostic Radiology and Nuclear Medicine, Rush University Medical Center, Chicago, IL, USA; c Department of Preventive Medicine, Northwestern University, Feinberg School of Medicine, Chicago, IL, USA; d Department of Medical Imaging, Ann & Robert H. Lurie Children’s Hospital of Chicago, Chicago, IL, USA

**Keywords:** Brown adipose tissue, adiposity, metabolism

## Abstract

**Objective**: To explore relationships between PET/CT characteristics of cold-activated brown adipose tissue (BAT), measures of adiposity and metabolic markers.

**Methods**: We conducted a post-hoc analysis of a study which utilized PET/CT to characterize BAT. 25 men ages 18–24 (BMI 19.4 to 35.9 kg/m^2^) were studied. Fasting blood samples were collected. Body composition was measured using DXA. An individualized cooling protocol was utilized to activate BAT prior to imaging with PET/CT.

**Results**: There was an inverse relationship between fasting serum glucose and BAT volume (r = −0.40, p = 0.048). A marginally significant inverse relationship was also noted between fasting glucose and total BAT activity (r = −0.40, p = 0.05). In addition, a positive correlation was observed between serum FGF21 and SUV_max_ (r = 0.51, p = 0.01). No significant correlations were noted for measures of BAT activity or volume and other indicators of adiposity or glucose metabolism.

**Conclusions**: The presence of active BAT may be associated with lower fasting glucose in young men. BAT activity may also be correlated with levels of FGF21, suggesting that BAT may lower glucose levels via an FGF21 dependent pathway. Further studies are needed to clarify mechanisms by which BAT may impact glucose metabolism.

## Introduction

More than a third (38.9%) of U.S. adults are obese, with a body mass index (BMI) of 30 kg/m^2^ or greater [1]. Many factors play a role in the development of obesity, including lifestyle factors leading to an imbalance of energy expenditure and energy intake, along with genetic factors that may drive increased weight gain when intake exceeds expenditure. Although caloric restriction can be an effective intervention for obesity, weight loss produces a significant reduction in energy expenditure, beyond what would be expected due to the loss of body mass. This reduction in energy expenditure (‘metabolic adaptation’ to weight loss) can make maintenance of a lower body weight more difficult. Therefore, interventions which increase energy expenditure may be useful for long-term obesity management as well as prevention. In recent years, brown adipose tissue (BAT) has emerged as a potential target of interest in this regard. In rodents, BAT is responsible for modulating energy expenditure and generating heat with overfeeding and cold exposure [2]. Data in humans suggest that individuals with active BAT as visualized on 18F-fluoro-2-deoxy-glucose (^18^F-FDG) positron emission tomography and computed tomography (PET/CT) have a greater rise in energy expenditure after cold exposure (cold-induced thermogenesis) and food intake (diet-induced thermogenesis) compared to those without active BAT [3–5]. Studies in humans and mice suggest that BAT may improve insulin sensitivity, glucose homoeostasis, modulate energy expenditure and decrease weight gain [1,4,6,7]. BAT activity is also lower in obese compared to lean individuals, though it is not yet known whether this is a cause or consequence of obesity [8–10].

Though the mechanism of BAT-related improvement in glucose homoeostasis has not been fully elucidated, studies suggest that paracrine or endocrine actions of interleukin 6 (IL-6) or fibroblast growth factor 21 (FGF21) may contribute to the favourable metabolic changes observed [4,6,8,11]. While the liver is the main site of FGF21 production, sympathetic activation during cold exposure stimulates BAT to secrete FGF21 as well as IL-6 [12,13]. In a study by Lee and colleagues, FGF21 secretion during mild cold exposure was over 6-fold higher in adults with BAT than in those without BAT [14]. In rodents, FGF21 exerts both acute and chronic metabolic effects. Acutely, FGF21 lowers plasma glucose level and improves insulin sensitivity through CNS signalling to BAT [15,16]. Chronic FGF21 administration to rodents increases energy expenditure and decreases body weight [17]. Trials of FGF21 analogues in humans are limited and the results have been mixed. In some studies, subjects with type 2 diabetes experienced significant weight loss and improvement in fasting insulin; however, no improvements in fasting glucose have been demonstrated [18,19]. On the other hand, improvements in both glucose metabolism and insulin sensitivity have been observed after 5 hours of mild cold exposure in BAT-positive but not BAT-negative men [4]. In this study, subjects with BAT also had greater increases in circulating FGF21 than those without BAT.; Furthermore, in an elegant proof-of-concept study from Stanford et al., mice that received transplanted BAT from age and sex-matched donors had significant increases in insulin sensitivity and five-fold higher levels of FGF21 levels compared to sham-treated mice twelve weeks after transplant [6]. When BAT was transplanted from IL-6 knockout mice, however, improvements in glucose homoeostasis were lost and FGF21 levels did not increase, demonstrating the importance of IL-6 in regulating BAT-related metabolic improvements [6]. It is unknown whether IL-6 has direct effects or if its metabolic benefits are derived from induction of FGF21. In a cold acclimatization study in humans, adiponectin levels increased while leptin levels decreased; changes in both adipokines were negatively correlated with changes in BAT activity [14]. It remains unclear whether these adipokines play a role in mediating BAT-related changes in glucose homoeostasis.

In adults, depots of fat in the supraclavicular regions contain a mixture of brown and white adipocytes, termed brite (brown-in-white) or beige fat [20–22]. BAT activation is regulated by the sympathetic nervous system, which can be activated with cold and suppressed by fasting [23,24]. BAT, which contains type II deiodinase (D2), can also be activated by thyroid hormone signalling [25,26]. ^18^F-FDG PET/CT after mild cold exposure is the imaging standard used to detect metabolically active BAT [27–29].

The purpose of this analysis was to explore the relationships between cold-activated BAT visualized on PET/CT, measures of adiposity, and metabolic markers (fasting glucose, insulin, thyroid hormone, FGF21, IL-6, adiponectin and leptin) in young men. We hypothesized that men with obesity would have less active BAT compared to lean men. We also expected that participants with more activated BAT would have a favourable metabolic profile with lower fasting glucose along with higher FGF21, IL-6, and adiponectin levels.

## Methods

### Subjects

A post-hoc analysis was conducted using data from a cross-sectional study examining the utility of magnetic resonance imaging (MRI) compared to PET/CT to image BAT among young men with varying BMIs [30]. The study was approved by the Institutional Review Board and subjects provided written informed consent for participation. Twenty-seven men 18–24 years old with BMI ranging 18.5 to 39.9 kg/m^2^ were recruited. To avoid any potential effects of ageing and the menstrual cycle on metabolic markers and BAT activity, and to reduce the number of participants needed for this exploratory study, only young men were recruited.

Exclusion criteria for subjects included: haemoglobin A1c (HbA1c) ≥ 7.0% or fasting plasma glucose > 150 mg/dL (since hyperglycaemia may impact FDG-PET signal intensity), use of diabetes medication, presence of any medical condition that affects energy metabolism, use of medications affecting brown fat activity, and contraindications to MRI. As fluctuations in weight may influence BAT activity and metabolic markers, participants were required to be weight stable (no more than 3% weight change in the 3 months prior to the study) and to be within 3% of their highest body weight [31].

### Study protocol

During a screening visit (a mean of 15 +/ –12 days) prior to imaging visits, subjects underwent a physical exam and fasting lab draw including measurement of fasting glucose, insulin, HbA1c, triglycerides, total cholesterol, low-density lipoproteins (LDL), high-density lipoproteins (HDL), thyroid-stimulating hormone (TSH) and free thyroxine (fT4), FGF21, IL-6, adiponectin and leptin. Fasting glucose and lipids were measured with a Beckman Coulter AU5800 Chemistry Analyser, using the hexokinase G6PDH method (glucose) and enzymatic method using oxidase and esterase (total cholesterol, TG and HDL; LDL was calculated). HbA1c measurement was done on a Trinity Premier Hb9210 analyser, using the boronate affinity method and HPLC. TSH and fT4 were determined on a Beckman DXi 800 analyser, using a sequential two-step immunoenzymatic sandwich assay. Insulin was measured using a Roche e411 analyser. FGF21, IL-6, leptin, and adiponectin were measured by ELISA using the appropriate kits from Millipore (#EZHFGF21-19K, #EZHIL6, #EZHL-80SK, and #EZHADP-61K, respectively). Insulin resistance was evaluated using the Homoeostasis Model Assessment (HOMA2 IR; using the HOMA2 Calculator from the University of Oxford, Oxford, UK) [32]. Participants arrived for separate imaging visits after fasting for at least 5 hours and abstaining from alcohol, caffeine, energy drinks and strenuous activity for the previous 24 hours. Capillary blood glucose was checked on arrival to ensure glucose level ≤ 150 mg/dL. Adiposity was determined using dual energy X-ray absorptiometry (DXA) scan (Lunar iDXA, GE Healthcare, Milwaukee, WI). Based on DXA data, fat mass index (FMI) was calculated as the ratio of fat mass in kilograms to height in metres squared; percentage of body fat was calculated as the ratio of fat mass to total body mass.

An individualized cooling protocol was used for BAT activation. Each participant was wrapped in a water-infused suit connected to a temperature control system (CritiCool® System and Universal ThermoWrap, Mennen Medical, Israel/Belmont Medical Technologies). The torso, upper arms and legs were wrapped with the suit, but head, hands, and feet were not wrapped. The water temperature in the system was initially set to 13°C to cool down the subject until the onset of shivering, and then the system temperature was increased by 1°C every 4 minutes until the subject stopped shivering. Once the system temperature was stable for at least 15 minutes and the subject was no longer shivering, this was designated as the individual’s unique non-shivering thermogenesis (NST) temperature. Subsequently, cold-activated BAT was visualized by ^18^F-FDG PET/CT (GE Discovery 690 VCT, GE Healthcare). ^18^F-FDG was administered at a dose of 0.075 mCi/kg, with a maximum dose of 10.0 mCi. The skull base to diaphragm was imaged initially with a low dose CT scan (120 kV, 30 mAs per slice and 64 × 0.625 mm of collimation) followed by PET scan. MatLab toolbox of Medical Image Reader and Viewer (MathWorks, Natick, MA) was used to analyse PET and CT images. Activated BAT from the neck to axilla was identified as increased FDG uptake with standardized uptake value normalized to total body mass (SUV_bm_) > 2.0 g/mL and −200 and −10 Hounsfield unit (HU) on CT. Regions-of-interest of metabolically active BAT were defined manually on each axial slice of the fused PET/CT images. The peak metabolic activity adjusted for body weight, maximal SUV (SUV_max_ in g/mL), average SUV within the anatomical region defined as BAT (SUV_mean_ in g/mL), and BAT volume (in mL) were measured. SUV_lean_ was calculated as the product of SUV_bm_ and lean mass divided by total body mass. Total BAT activity in KBq was calculated as the product of SUV_mean_ in g/mL, BAT volume (in mL) and the ratio of total FDG dose to the subject’s weight (in KBq/g).

### Statistical analysis

Statistical analysis was performed using SAS 9.4 (Cary, NC). For normally distributed variables, data are expressed as mean and standard deviation. Data that were not normally distributed are reported as median (interquartile range). Pearson and Spearman’s rank correlations were determined to relate measures of activated BAT to adiposity (BMI, FMI, and waist circumference) and biomarkers, such as fasting glucose and insulin. ANOVA and Kruskal-Wallis tests were used to determine if BAT measures differed among various racial groups. Independent t-tests and Wilcoxon tests were used to determine if BAT measures differed by ethnicity. The threshold for statistical significance was set at an α-error of 0.05. Since this is an exploratory study with the intent to generate hypotheses, Bonferroni correction for multiple comparisons was not done.

## Results

Data from a total of 25 men were included in the current analysis; 2 participants were missing imaging data and were therefore excluded. Study participants’ characteristics including demographics, anthropometric and lab data are summarized in Table 1. 52% of participants were white non-Hispanic men. Participants’ average age was 21 years. Participants’ BMI ranged from 19.4 to 35.9, with an average BMI of 25.2 kg/m^2^. 15 subjects had a normal BMI (18.5–24.9 kg/m^2^), 6 subjects had a BMI in the overweight range (25–29.9 kg/m^2^), and 4 subjects had a BMI ≥ 30 kg/m^2^. Subjects’ median body fat percentage was 21.2%; 8 men had < 15% body fat and 9 had > 25% body fat. Average fasting laboratory values including glucose, HbA1c, and lipid panel were in the normal range. All participants also had normal TSH and fT4 levels (data not shown). Twenty-one of the 25 subjects had evidence of active BAT on PET/CT images. Median total BAT activity was 362.9 kBq and BAT volume was 37.6 mL (Table 2). BAT imaging characteristics did not differ significantly by race or ethnicity (data not shown).

**Table 1.** Participant characteristics

Characteristic	N(%)	
Race		
Asian	6 (24%)	
Black/African American	4 (16%)	
White	15 (60%)	
Ethnicity		
Hispanic/Latino	4 (16%)	
Non Hispanic/Latino	21 (84%)	
	Mean (SD)	Range (min-max)
Age (years)	21.1 (1.8)	18 – 24
Weight (kg)	80.5 (17.4)	56.5–118.9
BMI (kg/m^2^)	25.2 (4.8)	19.4–35.9
	Median (25th, 75th percentile)	
Waist circumference (cm)*	82.5 (78.4, 99.5)	70 – 120
Body fat (%)*	21.2 (14.7, 27.5)	9.4–40.2
	Mean (SD)	
Glucose (mg/dL)	85.6 (6.0)	77 – 104
Insulin (uU/mL)	9.5 (5.6)	3.4–27.5
HbA1c (%)	5.3 (0.2)	4.9–5.7
HDL (mg/dL)	49.4 (14.8)	32 – 99
LDL (mg/dL)	86.9 (23.6)	53 – 136
Triglycerides (mg/dL)	74.9 (29.3)	34 – 129
Total cholesterol (mg/dL)	149.6 (28.6)	109 – 205
	Median (25th, 75th percentile)	
FGF21 (pg/mL)*	77.7 (43.2, 120.1)	11.0–204.7
Leptin (ng/mL)*	2.9 (1.2, 7.3)	0.4–42.2

Data not normally distributed, median presented rather than mean.

**Table 2.** Brown adipose tissue (BAT) characteristics on PET/CT imaging

Variable	Mean (SD)	min, max
SUV_mean_ (g/mL)	3.56 (1.93)	0, 7.94
SUV_max_ (g/mL)	10.52 (7.31)	0, 27
	Median (25th, 75th percentile)	
Total BAT activity (KBq)	362.9 (93.7, 1045.2)	0, 3904.3
BAT volume (mL)	37.6 (10.6, 86.7)	0, 178.2

*Data not normally distributed, median presented rather than mean.

**Figure 1.**
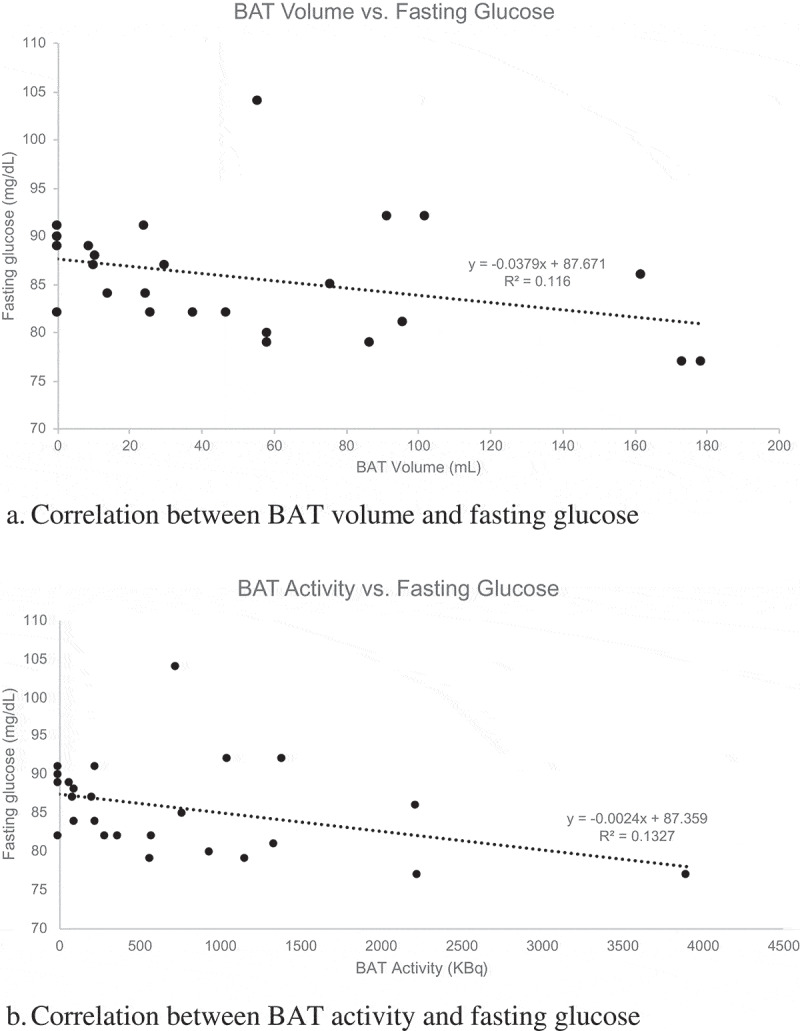
Correlation between BAT characteristics on PET/CT imaging and fasting glucose

**Figure 2.**
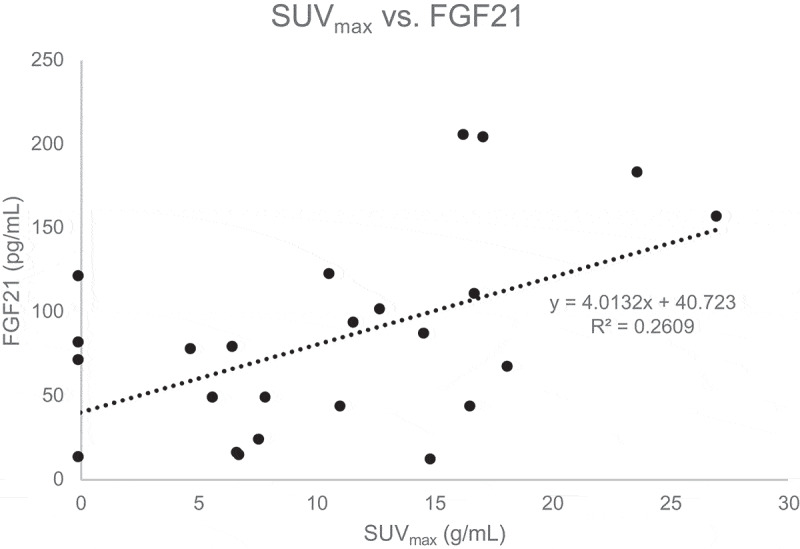
Correlation between SUV_max_ and FGF21

Correlations between BAT and measures of adiposity and metabolic parameters are shown in Table 3. There were no significant correlations between measures of adiposity including BMI, FMI, waist circumference and BAT. As shown in Figure 1, fasting serum glucose at the screening visit was inversely related to BAT volume (r = −0.40, p = 0.048) and marginally inversely associated with total BAT activity (r = −0.40, p = 0.05). No statistically significant correlations between BAT measures and other measures of glucose homoeostasis such as insulin, HOMA2-IR, or HbA1c were observed. Serum FGF21 was directly correlated with SUV_max_ (r = 0.51, p = 0.01, Figure 2). There was also a nonsignificant trend for a positive correlation between IL-6 and SUV_max_ (r = 0.37, p = 0.07).

**Table 3.** Correlations between SUV_mean_, SUV_max_, total BAT activity, BAT volume and measures of adiposity, and metabolic markers

Characteristic	SUV_mean_ r (p-value)^†^	SUV_max_ r (p-value)^†^	Total BAT activity r (p-value)^‡^	BAT volume r (p-value)^‡^
Age	0.08 (0.69)	−0.03 (0.88)	−0.03 (0.90)	−0.03 (0.90)
BMI	−0.22 (0.30)	−0.18 (0.38)	−0.10 (0.63)	−0.13 (0.53)
FMI	−0.19 (0.38)	−0.13 (0.53)	0.02 (0.92)	0.01 (0.97)
Waist Circumference	−0.15 (0.46)	−0.10 (0.64)	−0.05 (0.80)	−0.08 (0.71)
Glucose	−0.30 (0.14)	−0.38 (0.06)	**−0.40 (0.05)**	**−0.40 (0.048)**
HbA1c	−0.04 (0.85)	−0.09 (0.68)	−0.09 (0.68)	−0.10 (0.64)
Insulin	0.01 (0.96)	0.05 (0.80)	0.07 (0.76)	0.05 (0.81)
HOMA2-IR	0.00 (0.99)	0.04 (0.84)	0.07 (0.73)	0.06 (0.79)
Leptin	−0.33 (0.12)	−0.26 (0.22)	−0.04 (0.85)	−0.08 (0.71)
Adiponectin	0.09 (0.67)	0.11 (0.58)	0.24 (0.25)	0.21 (0.32)
FGF21	0.35 (0.09)	**0.51 (0.01)**	0.31 (0.14)	0.29 (0.17)
IL-6	0.34 (0.09)	0.37 (0.07)	−0.17 (0.42)	−0.11 (0.59)
TSH	0.09 (0.67)	0.05 (0.80)	0.13 (0.54)	0.15 (0.48)
fT4	0.06 (0.77)	−0.13 (0.54)	−0.13 (0.55)	−0.11 (0.61)

†Pearson correlations.

‡Spearman’s rank correlations.

In light of the potential relationship between FGF21 and BAT, we examined associations between FGF21 and measures of adiposity. Higher FGF21 levels were associated with higher FMI (r = 0.46, p = 0.02) and percentage of body fat (r = 0.50, p = 0.01).

In this cohort, there were no significant correlations between ambient temperature and PET/CT measures of BAT activity or volume (data not shown); this analysis was undertaken since it is known that seasonal changes in temperature can affect BAT activity.

## Discussion

In this cohort of generally healthy young men, a majority (84%) had active BAT on PET/CT imaging after mild cold exposure. BAT characteristics did not differ by race or ethnicity. Previous studies have demonstrated lower BAT activity among those with obesity compared to lean individuals [8–10]. In this study, however, there were no significant relationships observed between measures of active BAT and measures of adiposity including BMI, FMI and waist circumference. This discrepancy may in part be a result of the young age of our study participants or our small sample size. Prior research has suggested that BAT activity declines with age and that these age-related decreases in BAT activity may contribute to increased adiposity in later life [33]. Therefore, in a group of young subjects of similar age (as in this study), BAT activity may not be associated with adiposity. Further research would be needed to determine the relationships between BAT activity, adiposity, and ageing.

Both animal and human studies have demonstrated that BAT plays an important role in glucose homoeostasis and that it may protect against the development of diabetes [4,6]. In a study of 12 healthy male participants, Chondronikola et al demonstrated that in addition to increasing resting energy expenditure, cold-activated BAT significantly increased glucose disposal and whole-body insulin sensitivity [4]. The current study also supports the physiologic role of BAT in glucose disposal; participants with more BAT had lower fasting serum glucose levels, days prior to cold exposure. Data from the current study does not demonstrate any notable associations between BAT and insulin resistance as measured by HOMA2-IR, a measure of whole-body insulin resistance calculated from fasting labs. However, this calculation does not provide information about the post-prandial state and cannot assess peripheral insulin sensitivity; a mixed meal test or hyperinsulinemic euglycemic clamp (as used by Chondronikola et al) would be required.

Hormones and cytokines including fT4, FGF21, IL-6, leptin and adiponectin have been associated with changes in active BAT [6,21,34]. Circulating FGF21, released largely by the liver with some contribution from BAT, has been shown to increase *GLUT1* expression in adipose tissue and improve insulin sensitivity [12,35]. Stanford et al. demonstrated that IL-6 is a necessary component in BAT-related improvement in glucose homoeostasis [6]. In the current study, FGF21 was directly associated with one of the imaging parameters, SUV_max_. Similarly, there was a nonsignificant trend for an association between IL-6 and SUV_max_. Since fasting glucose levels were drawn before participants were exposed to cold temperatures, the association between BAT imaging parameters and glycaemia may reflect prior BAT activation. Taken together, these results support the current understanding of active BAT as an endocrine organ which may improve glucose homoeostasis in an FGF21-dependent manner. Postulated mechanisms underlying BAT-related improvement in metabolism and glucose homoeostasis include: paracrine and endocrine actions of FGF21 and IL-6, acute increases in glucose and fatty acid use by metabolically active BAT depots, and increased thermogenesis leading to changes in body composition including a decrease in body fat [6,8]. We found that circulating FGF21 levels were directly associated with measures of adiposity in this study cohort. Despite this apparent paradox (given the metabolic benefits conferred by FGF21), our findings are consistent with those of several others demonstrating a positive association between FGF21 levels and whole-body and visceral fat mass [36–41]. It is hypothesized that individuals with obesity may be resistant to FGF21, whereby circulating levels are elevated but target tissues do not respond to the effects of FGF21. While the FGF21-resistant state has not been definitively proven, a study by Berti et al found that serum FGF21 concentrations were higher in metabolically unhealthy compared to metabolically healthy obese individuals [42].

In the present study, which involved subjects with normal thyroid hormone levels, no significant correlations were observed between TSH or fT4 and subsequent BAT activity. Thyroid hormone plays a role in differentiation and activation of BAT, acting synergistically with the sympathetic nervous system to induce UCP1 expression and thermogenesis in BAT [43]. Much of the recent literature examining the relationship between thyroid hormone and BAT activity has focused on patients with overt hypo- or hyperthyroidism, yielding mixed results [43]. However, two recent studies which have explored the relationship between thyroid hormone levels and BAT activity in euthyroid subjects reported an inverse relationship between BAT activity and free T4 level, and one of these studies also found an inverse relationship between BAT activity and free T3 [44,45]. Additional research is needed to understand whether and how thyroid hormone levels within the normal range influence an individual’s BAT volume and activity.

In a prior study, chronic mild cold acclimation (and the resulting increase in BAT) has been shown to be accompanied by an increase in circulating adiponectin and a decrease in leptin [34]. In the current study, however, we saw no significant relationships between active BAT and leptin or adiponectin levels.

Strengths of this study include the use of an individualized cooling protocol, PET/CT imaging of BAT and DXA imaging of body composition, as well as the wide range of body mass indices among subjects. Subjects were all young men, which limited the potential confounding effects of age and gender on BAT activity and metabolic markers. Unfortunately, as a consequence, the study results may be less generalizable to women and older men. Another limitation of the study was a relatively small sample size and the lack of repeated laboratory tests after cold exposure/BAT activation. Furthermore, all analysis reported here were completed post-hoc as the primary intent of the study was to compare imaging characteristics of BAT with PET/CT and MRI.

## Conclusion

In this cohort of young men with varying body compositions, there were no associations between measures of adiposity and active BAT. BAT volume and activity were inversely related to fasting serum glucose, suggesting active BAT may have beneficial effects on glucose metabolism. Serum FGF21 levels were directly correlated with active BAT; together, these findings suggest that FGF21 may be involved in BAT-related improvements in glucose metabolism. Additional studies in humans are needed to clarify these potential relationships and mechanisms of action. If BAT is found to have a beneficial effect on glucose metabolism, interventions aimed at increasing BAT activity may prove useful in diabetes prevention or treatment.
